# Machine Learning methods for Quantitative Radiomic Biomarkers

**DOI:** 10.1038/srep13087

**Published:** 2015-08-17

**Authors:** Chintan Parmar, Patrick Grossmann, Johan Bussink, Philippe Lambin, Hugo J. W. L. Aerts

**Affiliations:** 1 Departments of Radiation Oncology; 2 Radiology, Dana-Farber Cancer Institute, Brigham and Women’s Hospital, Harvard Medical School, Boston, MA, USA; 3 Radiation Oncology (MAASTRO), Research Institute GROW, Maastricht University, Maastricht, the Netherlands; 4 Machine Intelligence Unit, Indian Statistical Institute, Kolkata, India; 5 Department of Biostatistics & Computational Biology, Dana-Farber Cancer Institute, Boston, MA, USA; 6 Department of Radiation Oncology, Radboud University Medical Center, Nijmegen, the Netherlands

## Abstract

Radiomics extracts and mines large number of medical imaging features quantifying tumor phenotypic characteristics. Highly accurate and reliable machine-learning approaches can drive the success of radiomic applications in clinical care. In this radiomic study, fourteen feature selection methods and twelve classification methods were examined in terms of their performance and stability for predicting overall survival. A total of 440 radiomic features were extracted from pre-treatment computed tomography (CT) images of 464 lung cancer patients. To ensure the unbiased evaluation of different machine-learning methods, publicly available implementations along with reported parameter configurations were used. Furthermore, we used two independent radiomic cohorts for training (n = 310 patients) and validation (n = 154 patients). We identified that Wilcoxon test based feature selection method WLCX (stability = 0.84 ± 0.05, AUC = 0.65 ± 0.02) and a classification method random forest RF (RSD = 3.52%, AUC = 0.66 ± 0.03) had highest prognostic performance with high stability against data perturbation. Our variability analysis indicated that the choice of classification method is the most dominant source of performance variation (34.21% of total variance). Identification of optimal machine-learning methods for radiomic applications is a crucial step towards stable and clinically relevant radiomic biomarkers, providing a non-invasive way of quantifying and monitoring tumor-phenotypic characteristics in clinical practice.

‘Precision oncology’ refers to the customization of cancer care, where practices and/or therapies are being tailored to individual patients. Such customization process can maximize the success of preventive and therapeutic interventions with minimum side effects. Most of the precision oncology related research has centered on the molecular characterization of tumors using genomics based approaches, which require tissue extraction by tumor biopsies. Although several genomics based approaches have successfully been applied in clinical oncology^1^, there are inherent limitations to biopsy based assays. Tumors are spatially and temporally heterogeneous, and repeated tumor biopsies, which increase the risk for a patient, are often required to capture the molecular heterogeneity of tumors. These ethical and clinical challenges related to biopsy-based assays, can be addressed by medical imaging, which is a routine practice for cancer diagnosis and staging in clinical oncology. Unlike biopsies, medical imaging is non-invasive and can provide information regarding the entire tumor phenotype, including the intra-tumor heterogeneity. Furthermore, recent advances in high-resolution image acquisition machines and computational hardware allow the detailed and efficient quantification of tumor phenotypic characteristics. Therefore, medical imaging provides unprecedented opportunities for precision oncology.

“Radiomics”, an emerging and promising field, hypothesizes that medical imaging provides crucial information regarding tumor physiology, which could be exploited to enhance cancer diagnostics^2^. It provides a comprehensive quantification of tumor phenotypes by extracting and mining large number of quantitative imaging features^3^. Several studies have investigated various radiomic features in terms of their prognostic or predictive abilities and reliability across different clinical settings^4^,^5^,^6^,^7^,^8^,^9^,^10^. Different studies have shown the discriminating capabilities of radiomic features for the stratification of tumor histology^6^, tumor grades or stages^11^, and clinical outcomes^8^,^12^,^13^. Moreover, some studies have reported the association between radiomic features and the underlying gene expression patterns^8^,^14^,^15^.

“Machine-learning” can be broadly defined as computational methods/models using experience (data) to improve performance or make accurate predictions^16^. These programmable computational methods are capable of “learning” from data and hence can automate and improve the prediction process. Predictive and prognostic models with high accuracy, reliability, and efficiency are vital factors driving the success of radiomics. Therefore, it is essential to compare different machine-learning models for radiomics based clinical biomarkers. Like any high-throughput data-mining field, radiomics also underlies the curse of dimensionality^17^, which should be addressed by appropriate feature selection strategies. Moreover, feature selection also helps in reducing overfitting of models (increasing the generalizability). Thus, in order to reduce the dimensionality of radiomic feature space and enhance the performance of radiomics based predictive models, different feature selection methods^18^ should be thoroughly investigated. However, as radiomics is an emerging research field, most of the published studies have only assessed the predictive capabilities of radiomic features without putting much emphasis on the comparison of different feature selection and predictive modeling methods. Only few recent studies have investigated the effect of different feature selection and machine learning classification methods on radiomics based clinical predictions^19^,^20^, but with limited sample sizes. Furthermore, these studies lacked independent validation of the results, which may restrict the generalizability of their conclusions.

In this study, we investigated a large panel of machine-learning approaches for radiomics based survival prediction. We evaluated 14 feature selection methods and 12 classification methods in terms of their predictive performance and stability against data perturbation. These methods were chosen because of their popularity in literature. Furthermore, publicly available implementations along with reported parameter configurations were used in the analysis, which ensured an unbiased evaluation of these methods. Two independent lung cancer cohorts were used for training and validation, with in total image and clinical outcome data of 464 patients. Feature selection and predictive modeling are considered as the important building blocks for high throughput data driven radiomics. Therefore, our investigation could help in the identification of optimal machine-learning approaches for radiomics based predictive studies, which could enhance the applications of non-invasive and cost-effective radiomics in clinical oncology.

## Methods

### Radiomic Features

A total of 440 radiomic features were used in the analysis. These radiomic features quantified tumor phenotypic characteristics on CT images and are divided into four feature groups: I) tumor intensity, II) shape, III) texture and IV) wavelet features. Tumor intensity based features estimated the first order statistics of the intensity histogram, whereas shape features described the 3D geometric properties of the tumor. Textural features, derived from the gray level co-occurrence (GLCM)^21^ and run length matrices (GLRLM)^22^, quantified the intra-tumor heterogeneity. These textural features were computed by averaging their values over all thirteen directions. Wavelet features are the transformed domain representations of the intensity and textural features. These features were computed on different wavelet decompositions of the original image using a coiflet wavelet transformation. Matlab R2012b (The Mathworks, Natick, MA) was used for the image analysis. Radiomic features were automatically extracted by our in-house developed radiomics image analysis software, which uses an adapted version of CERR (Computational Environment for Radiotherapy Research)^23^ and Matlab for the preprocessing of medical images. Mathematical definitions of all radiomic features, as well as the extraction methods, were previously described^8^.

### Datasets

In this study, we employed two NSCLC cohorts from the two different institutes of Netherlands: (1) Lung1:422 NSCLC patients treated at MAASTRO Clinic in Maastricht. (2) Lung2:225 NSCLC patients treated at Radboud University Medical Center in Nijmegen. CT-scans, manual delineations and clinical data were available for all included patients. More details on the included datasets are described in Supplementary-A. We dichotomized the censored continuous survival data using a cutoff time of 2 years. The patients who lived beyond the cutoff time were labeled as 1, whereas the deceased ones were labeled as 0. The objective of the study was to stratify patients into these two labeled survival classes. Two-years is considered as a relevant survival time for NSCLC patients and several other studies have designed their prediction models using a survival cutoff of 2 years^24^,^25^,^26^. We excluded the patients, which were followed for less than 2 years. It resulted in 310 patients in training cohort (Lung1) and 154 patients in validation cohort (Lung2). All the features were normalized using Z-score normalization.

### Feature Selection Methods

Fourteen feature selection methods based on filter approaches were used in the analysis (Fisher score (FSCR), Relief (RELF), T-score (TSCR), Chi-square (CHSQ), Wilcoxon (WLCX), Gini index (GINI), Mutual information maximization (MIM), Mutual information feature selection (MIFS), Minimum redundancy maximum relevance (MRMR), Conditional infomax feature extraction (CIFE), Joint mutual information (JMI), Conditional mutual information maximization (CMIM), Interaction capping (ICAP), Double input symmetric relevance (DISR)). In order to improve the readability of this manuscript, we have defined all the acronyms related to feature selection methods in Table 1. We chose these methods mainly because of their popularity in literature, simplicity and computational efficiency. Furthermore, publicly available implementations were readily available for these methods^27^,^28^, which increases their reusability. Filter methods are feature-ranking methods, which rank the features using a scoring criterion. All filter based feature selection methods can be divided into two categories: univariate methods and multivariate methods. In case of univariate methods, the scoring criterion only depends on the feature relevancy ignoring the feature redundancy, whereas multivariate methods investigate the multivariate interaction within the features and the scoring criterion is a weighted sum of feature relevancy and redundancy. Feature relevancy is a measure of feature’s association with the target/outcome variable, whereas feature redundancy is the amount of redundancy present in a particular feature with respect to the set of already selected features. Further description regarding the theoretical formulation of feature selection problem and each of the used feature selection methods can be obtained from Supplementary-B online.

### Classifiers

In machine-learning, the classification is considered as a supervised learning task of inferring a function from labeled training data^16^. The training data consists of a set of examples, where each example is represented as a pair of an input vector (features) and a desired output value (target or category label). The classification algorithm (classifier) analyzes the training data and infers a hypothesis (function), which can be used for predicting the labels of unseen observations. Many classifiers belonging to different areas of computer science and statistics have been proposed in machine-learning literature^29^. In our study, we used 12 machine-learning classifiers arising from 12 classifier families (Bagging (BAG), Bayesian (BY), Boosting (BST), Decision trees (DT), Discriminant analysis (DA), Generalized linear models (GLM), Multiple adaptive regression splines (MARS), Nearest neighbors (NN), Neural networks (Nnet), Partial least square and principle component regression (PLSR), Random forests (RF), and Support vector machines (SVM)). The acronyms related to classifiers are defined in Table 1. All classifiers were implemented using R package caret^30^, which provides a nice interface to access many machine-learning algorithms in R. Furthermore, it also provides a user-friendly framework for training different machine-learning models. Classifiers were trained using the repeated (3 repeat iterations) 10 fold cross validation of training cohort (Lung1) and their predictive performance was evaluated in the validation cohort (Lung2) using area under ROC curve (AUC). We used parameter configurations that were previously defined by Fernandez-Delgado *et al.*^31^ in a comprehensive comparative study of 179 classifiers and 121 different datasets. We have listed the classification methods along with their parameters and corresponding R packages in Supplementary-C online.

## Analysis

### Predictive Performance of Feature Selection and Classification Methods

In order to investigate and compare different feature selection and classification methods, we created a three-dimensional parameter grid for the analysis. For each of the 14 feature selection methods, we incrementally selected features ranging from 5 up to 50, with an increment of 5 features (n = 5, 10, 15, 20, … , 50). These subsets of selected features were then evaluated by using each of the 12 machine-learning classifiers and area under ROC curves (AUC).

### Stability of Feature Selection and Classification Methods

In order to assess the stability of feature selection methods, we used a stability measure proposed by Yu *et al.*^32^ under the hard data perturbation settings^33^. We quantified the stability of a method as the similarity between the results obtained by the same feature selection method, when applied on the two non-overlapping partitions (of size N/2) of the training cohort (Lung1). To compute similarity between the two resultant feature sets, a weighted complete bipartite graph was constructed, where the two node sets corresponded to the two sets of selected features. The edge weights were assigned as the absolute Spearman correlation coefficient between the features at the nodes. We then applied the Hungarian algorithm^34^ to identify the maximum weighted matching between the two node sets, and then similarity (stability) was quantified as the final matching cost. For each feature selection method, we computed the stability 100 times using a bootstrap approach and reported the median ± std values in the results.

The empirical stability of a classifier was quantified using the relative standard deviation (RSD %) and a bootstrap approach. We first selected 30 representative features using the Wilcoxon based feature selection method WLCX and used them to compute the classifier stability. For each classification method, we trained the model on the subsampled training cohort (size N/2) and validated the performance on the validation cohort using AUC. Subsampling of the training cohort was done 100 times using a bootstrap approach. RSD is the absolute value of the coefficient of variation and is often expressed in percentage. Here, it was defined as


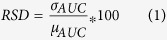


where 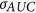 and 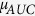 were the standard deviation and mean of the 100 AUC values respectively. It should be noted that higher stability in the case of classifiers corresponds to lower RSD values.

### Stability and Predictive Performance

In order to identify the highly reliable and accurate methods, we used the median values of AUC and stability as thresholds. We created two rank lists based on AUC & stability and cited the methods as highly accurate and reliable, which ranked in the top half of both the ranked lists. Feature selection methods having stability ≥0.735 (median stability of all feature selection methods) and AUC ≥ 0.615 (median AUC of all feature selection methods) are considered as highly reliable and accurate methods. Similarly, classification methods having RSD ≤ 5.97 (median RSD of all classifiers) and AUC ≥ 0.61 (median AUC of all classifiers) are considered as highly reliable and accurate ones.

### Experimental Factors Affecting the Radiomics Based Survival Prediction

There are three main experimental factors, which can potentially affect the prediction of radiomics based survival prediction: feature selection method, classification method and the number of selected features. Multifactor ANOVA was used to quantify the variability in AUC scores contributed by these factors and their interactions. In order to compare the variability contributed by each factor, the estimated variance components were divided by the total variance.

All the analysis was done using R software (R Core Team, Vienna, Austria) version 3.1.2 and Matlab R2012b (The Mathworks, Natick, MA) with Windows 7.

## Results

**Figure 1:**
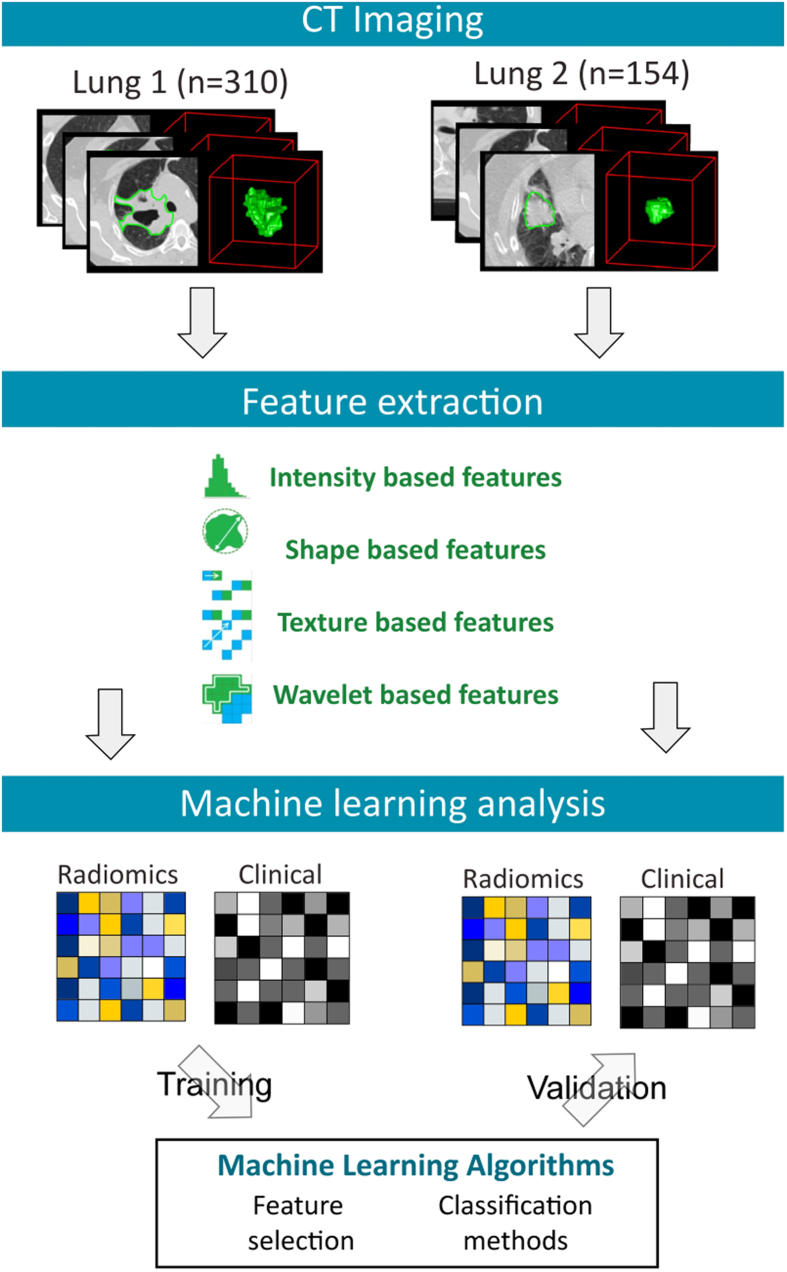
A total of 440 radiomic features were extracted from the segmented tumor regions of the pre-treatment CT images of 464 NSCLC patients. Feature selection and classification training was done using the training cohort Lung1 (n = 310), whereas Lung2 (n = 154) cohort was used as a validation cohort.

To investigate the machine-learning approaches for prognostic radiomic biomarkers, a total of 440 radiomic features were extracted from the segmented tumor regions of the pre-treatment CT images of two independent NSCLC cohorts. Feature selection and classification training was done using the training cohort Lung1 (n = 310 patients), whereas the validation cohort Lung2 (n = 154 patients) was used to assess the predictive performance [see Fig. 1].

### Predictive Performance of the Feature Selection and Classification Methods

**Figure 2:**
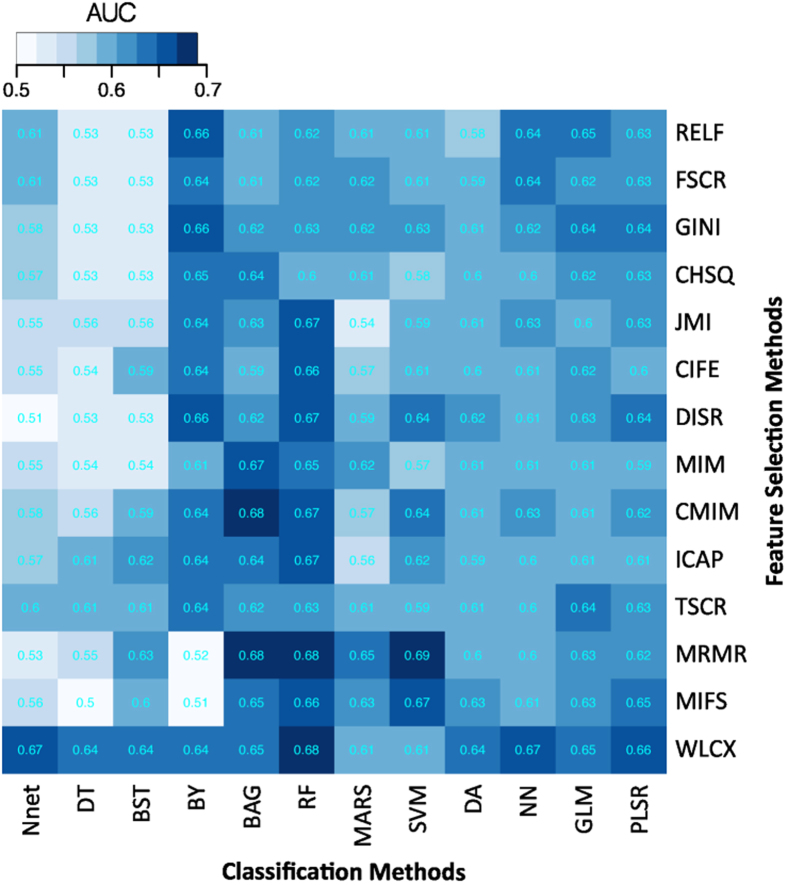
Heatmap depicting the predictive performance (AUC) of feature selection (in rows) and classification (in columns) methods. It can be observed that RF, BAG and BY classification methods and feature selection methods WLCX, MRMR and MIFS shows relatively high predictive performance in many cases.

Predictive performance of different feature selection and classification methods was assessed using the area under receiver operator characteristic curve (AUC). Figure 2 depicts the performance of feature selection (in rows) and classification methods (in columns) using 30 selected features, which are the 30 top ranked features, resulted in feature selection. For each classification method, there are 14 AUC values corresponding to the 14 different feature selection methods. We used a median of all 14 AUC values as a representative AUC of a classifier. Similarly, for each feature selection method, a median of 12 AUCs (corresponding to 12 classification methods) is used as a representative AUC. These representative AUC values for the classification and feature selection methods are given in Table 2. For classification methods, random forest (RF) displayed highest predictive performance (AUC: 0.66 ± 0.03) (median ± std), whereas decision tree (DT) (AUC: 0.54 ± 0.04) showed the lowest predictive performance. As far as feature selection methods are concerned, the Wilcoxon test based method WLCX showed highest predictive performance (AUC: 0.65 ± 0.02), whereas method CHSQ (AUC: 0.60 ± 0.03) and CIFE (AUC: 0.60 ± 0.04) had the lowest median AUCs. We repeated the above experiment by varying the number of selected features (range 5–50). Results corresponding to 10, 20, 40 and 50 representative (top ranked) features are reported in Supplementary Figures S1, S2, S3 and S4 online. Furthermore, median AUC values over each of the experimental factors (feature selection methods, classification methods and number of selected features) are depicted by the heatmaps in Supplementary Figures S5, S6 and S7 online. Here as well, random forest (RF) (classifier) and Wilcoxon test based method WLCX (feature selection) showed highest median AUCs in majority of cases.

### Stability of the Feature Selection and Classification Methods

We assessed the feature selection methods in terms of their stability against data resampling using the hard data perturbation settings^33^. We observed that MIM was the most stable method (stability = 0.94 ± 0.02) (median ± std) followed by RELIEF (stability = 0.91 ± 0.05) and WLCX (stability = 0.84 ± 0.05), whereas GINI (stability = 0.68 ± 0.10), JMI (stability = 0.68 ± 0.05), CHSQ (stability = 0.69 ± 0.09), DISR (stability = 0.69 ± 0.05) and CIFE (stability = 0.69 ± 0.05) showed relatively low stability [Table 2].

Empirical stability of classification methods was quantified using the relative standard deviation (RSD) and a bootstrap approach. We observed that BY was the most stable classification method (RSD = 0.86%) followed by GLM (RSD = 2.19%), PLSR (RSD = 2.24%) and RF (RSD = 3.52%). BST had the highest relative standard deviation in AUC scores (RSD = 8.23%) and hence the lowest stability among the classification methods. RSD (%) values corresponding to all 12 classifiers are reported in Table 2.

### Stability and Predictive Performance

**Figure 3:**
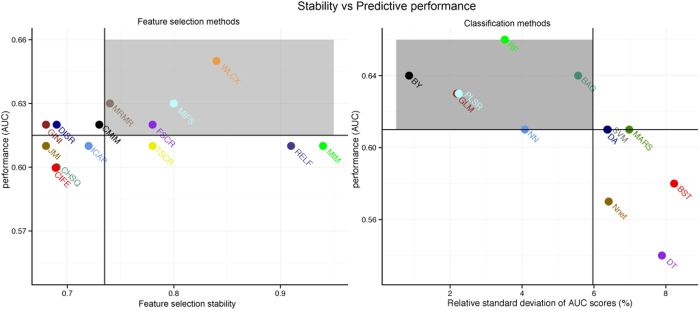
Scatterplots between the stability and predictive performance (AUC) of feature selection (FS) (Left) and classification methods (CF) (right). Feature selection methods having stability ≥0.735 (median stability of FS) and AUC ≥ 0.615 (median AUC of FS) are considered as highly reliable and predictive methods. Similarly, classification methods having RSD ≤ 5.97 (median RSD of CF) and AUC ≥ 0.61 (median AUC of CF) are considered as highly reliable and accurate ones. Highly reliable and predictive methods are displayed in a gray square region.

Scatterplots in Fig. 3 assesses the stability and prediction performance. It can be observed that feature selection methods WLCX (stability = 0.84 ± 0.05, AUC = 0.65 ± 0.02), MIFS (stability = 0.8 ± 0.03, AUC = 0.63 ± 0.03), MRMR (stability = 0.74 ± 0.03, AUC = 0.63 ± 0.03) and FSCR (stability = 0.78 ± 0.08, AUC = 0.62 ± 0.04) should be preferred as their stability and predictive performance was higher than the corresponding median values across all feature selection methods (stability = 0.735, AUC = 0.615). Similarly for classification methods, RF (RSD = 3.52%, AUC = 0.66 ± 0.03), BY (RSD = 0.86%, AUC = 0.64 ± 0.05), BAG (RSD = 5.56%, AUC = 0.64 ± 0.03), GLM (RSD = 2.19%, AUC = 0.63 ± 0.02), and PLSR (RSD = 2.24%, AUC = 0.63 ± 0.02), the stability and predictive performance was higher than the corresponding median values (RSD = 5.93%, AUC = 0.61).

### Experimental Factors Affecting the Radiomics Based Survival Prediction

**Figure 4:**
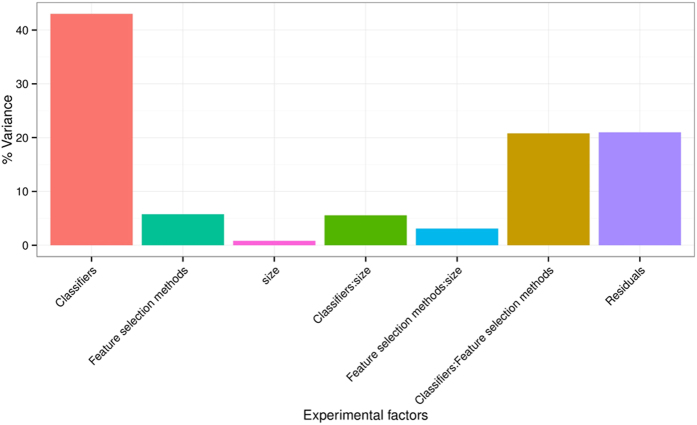
Variation of AUC explained by the experimental factors and their interactions. It can be observed that classification method was the most dominant source of variability. Size of the selected (representative) feature subset shared the least of the total variance.

To quantify the effects of the three experimental factors (feature selection methods, classification methods and the number of selected features), we performed multifactor analysis of variance (ANOVA) on AUC scores. We observed that all three experimental parameters and their interactions are the significant factors affecting the prediction performance [Fig. 4]. Classification method was the most dominant source of variability as it explained 34.21% of the total variance in AUC scores. Feature selection accounted for the 6.25%, whereas interaction of classifier & feature selection explained 23.03% of the total variation. Size of the selected (representative) feature subset only shared 1.65% of the total variance [Fig. 4].

## Discussion

Medical imaging is a routinely used and easily accessible source of information in clinical oncology. It serves as a non-invasive and cost-effective cancer diagnostic tool. Radiomics employs the medical imaging data for the customization of cancer care and hence adds a new and promising dimension to precision oncology^2^,^3^,^8^. Moreover, it can also capture the intra-tumor heterogeneity, which is often considered as an important biomarker in oncology^12^,^35^,^36^,^37^. A number of studies have built radiomics based predictive models for various clinical factors (tumor grades, survival outcomes, treatment response, etc.)^12^. For the successful realization of radiomics based predictive analyses, it is required to evaluate and compare different feature selection and predictive modeling methods, which was the primary objective of this study.

Various feature selection methods have been employed for high-throughput data mining problems^38^. In general, feature selection methods are categorized into three main categories: (1) filter methods (2) wrapper methods and (3) embedded methods. In this study, we investigated 14 different filter based approaches for radiomics based survival prediction. We only used filter-based approaches because they are computationally more efficient and less prone to overfitting than the wrapper and embedded methods^18^,^27^. Furthermore, unlike wrapper and embedded methods, filter methods are classifier independent. Thus, they allow separation of the modeling and feature selection component of the predictive analysis, which increases the generalizability of each component and hence the overall analysis.

We also investigated 12 machine-learning classification methods belonging to 12 different classifier families. Many classifiers have been proposed in the machine-learning literature. Theoretically speaking, these classifiers belong to different fields (classifier families) of computer science and statistics. Therefore, it could really be difficult to understand the underlying assumptions of each and every classifier and tune the parameters in an unbiased manner. The parameter tuning could be biased by user’s more (or lack of) expertise with some classifiers over the others. Usually, the studies, which propose a new classifier, only compare it to the reference classifiers of same family excluding the other classifier families. Even if classifiers belonging to different families are considered for comparison, these reference classifiers are usually implemented using simple tools and with limited parameter configurations while carefully tuning the proposed classifier. These could consequently bias the results in favor of the proposed classifiers^31^. In our study, we are not proposing any new classifier and we have used the same implementation tool (R package caret) for all the classifiers. Furthermore, to ensure unbiased usage of classifiers, we used parameter configurations that were previously defined by Fernandez-Delgado *et al.*^31^, in an exhaustive study of comparing 179 classifiers over 121 different datasets. These parameter configurations were selected from the literature and have been previously validated on a large number (121) of datasets belonging to different fields. Furthermore, in our study, the parameters were tuned using the repeated cross validation of training data only. Hence, our experimental design allowed us to evaluate different classification methods in an unbiased manner.

Our results show that the Wilcoxon test based feature selection method WLCX yields the highest predictive performance with the majority of classifiers. Interestingly, WLCX is a simple univariate method based on ranks, which does not take into account the redundancy of selected features during feature ranking. The majority of feature selection methods gave highest predictive performance when used with the random forest (RF) classifier. One could argue that with different parameter configurations, the performance of classification methods may improve further. An exhaustive parameter tuning could be investigated for evaluating the improvement of prediction performance. However, the required computational resources and high time complexity can hinder the exhaustive search. We expect that future radiomic studies focusing on different clinical outcomes and similar analysis framework could provide better understanding in this regard. A limited number of methods, which are consistently high performing across different radiomic studies, could be further assessed with an exhaustive parameter tuning. Nevertheless, It should be noted that random forests (RF) have displayed high predictive performance in several other biomedical and other domain applications as well^31^. These results indicate that choosing the WLCX feature selection method and/or RF classification method increases predictive performance in radiomics.

Results related to our stability analysis provide another dimension for choosing the feature selection and classification methods. Depending upon the applications, one may give importance to the predictive performance or stability and accordingly opt for the required method. Results related to multifactor ANOVA indicated that the classification method is the most dominant source of variation in the prediction performance (AUC) and hence should be chosen carefully. Size of the selected feature subset contributed the least in the total variation of AUC.

Only few studies have investigated and compared different feature selection and machine-learning modeling methods for radiomics based clinical predictions^19^,^20^. Recently, Hawkins *et al.*^19^ have compared four different feature selection and classification methods for CT based survival prediction of NSCLC patients. This study, however, was limited by the small cohort size as the final results were obtained on only 40 patients. Furthermore, it also lacked an independent validation of the results. On the contrary, two independent radiomic cohorts of sizes 310 and 154 patients were used in our analysis and an independent validation of the results was reported.

Our radiomic analysis is focused on the prediction of two-year patient survival in NSCLC patients. It provides an unbiased evaluation of different machine-learning methods of feature selection and classification. It could be considered as a reference for the future radiomics based predictive studies. Our results indicated that choosing Wilcoxon test based feature selection method WLCX and/or random forest (RF) classification method gives highest performance for radiomics based survival prediction. Furthermore, these methods also turned out reasonably stable against data perturbation and hence they could be preferred for radiomics based predictive studies. These results should be further tested in other radiomics based predictive studies, with different imaging modalities and in different cancer types.

It has been previously shown that for NSCLC patients, statistical models based on patient’s tumor and treatment characteristics provide significantly better predictions than the human expert^24^. Moreover, several other studies have highlighted the limitation of doctors’ prognostic capability for terminally ill cancer patients^39^,^40^,^41^. The predictions of human experts can suffer from inter-observer variability. On the contrary, statistical models could make the prediction system more deterministic if the parameter configurations and the training framework are fixed.

The potential clinical utility of radiomics based prognostic models has been stated in previous study^8^. With expanding radiomics cohorts and feature dimensions, we expect higher prediction performance in future radiomic studies. Furthermore, the integrative studies like radiomics-genomics in combination with standard clinical covariates could also improvise the prediction performance and further validate the utility of these methods in clinical practice. Overall, our analysis is a step forward towards the enhancements of radiomics based clinical predictions.

## Additional Information

**How to cite this article**: Parmar, C. *et al.* Machine Learning methods for Quantitative Radiomic Biomarkers. *Sci. Rep.* **5**, 13087; doi: 10.1038/srep13087 (2015).

## Supplementary Material

Supplementary Information

## Figures and Tables

**Table 1:** Table defining the acronyms related to the used feature selection and classification methods.

Classification method acronym	Classification method name	Feature Selection method acronym	Feature selection method name
Nnet	Neural network	RELF	Relief
DT	Decision Tree	FSCR	Fisher score
BST	Boosting	GINI	Gini index
BY	Bayesian	CHSQ	Chi-square score
BAG	Bagging	JMI	Joint mutual information
RF	Random Forset	CIFE	Conditional infomax feature extraction
MARS	Multi adaptive regression splines	DISR	Double input symmetric relevance
SVM	Support vector machines	MIM	Mutual information maximization
DA	Discriminant analysis	CMIM	Conditional mutual information maximization
NN	Neirest neighbour	ICAP	Interaction capping
GLM	Generalized linear models	TSCR	T-test score
PLSR	Partial least squares and prinicipal componenet regression	MRMR	Minimum redundancy maximum relevance
—	—	MIFS	Mutual information feature selection
—	—	WLCX	Wilcoxon

**Table 2:** Table describing the median values of AUC and stability for different Classification and Feature Selection methods.

Classification method	AUC	RSD %	Feature Selection method	AUC	Stability
Nnet	0.57 ± 0.04	6.41	RELF	0.61 ± 0.04	0.91 ± 0.05
DT	0.54 ± 0.04	7.89	FSCR	0.62 ± 0.04	0.78 ± 0.08
BST	0.58 ± 0.04	8.23	GINI	0.62 ± 0.04	0.68 ± 0.10
BY	0.64 ± 0.05	0.86	CHSQ	0.60 ± 0.04	0.69 ± 0.09
BAG	0.64 ± 0.03	5.56	JMI	0.61 ± 0.04	0.68 ± 0.05
RF	0.66 ± 0.03	3.52	CIFE	0.60 ± 0.03	0.69 ± 0.05
MARS	0.61 ± 0.03	6.98	DISR	0.62 ± 0.05	0.69 ± 0.05
SVM	0.61 ± 0.03	6.39	MIM	0.61 ± 0.04	0.94 ± 0.02
DA	0.61 ± 0.02	6.37	CMIM	0.62 ± 0.04	0.73 ± 0.04
NN	0.61 ± 0.02	4.08	ICAP	0.61 ± 0.03	0.72 ± 0.04
GLM	0.63 ± 0.02	2.19	TSCR	0.61 ± 0.02	0.78 ± 0.12
PLSR	0.63 ± 0.02	2.24	MRMR	0.63 ± 0.06	0.74 ± 0.03
—	—	—	MIFS	0.63 ± 0.06	0.8 ± 0.03
—	—	—	WLCX	0.65 ± 0.02	0.84 ± 0.05
