# Supplementary material for: Machine Learning methods for Quantitative Radiomic Biomarkers
Source: Sci Rep. 2015 Aug 17;5:13087. doi: 10.1038/srep13087 (PMC4538374; doi:10.1038/srep13087)
Supplement: Supplementary Information [file srep13087-s1.pdf]

Machine Learning methods for Quantitative Radiomic Biomarkers

Chintan Parmar[1,3,4*,#], Patrick Grossmann[1,5,#], Johan Bussink[6], Philippe Lambin[3], Hugo J.W.L. Aerts[1,2,5,*]

Departments of [1]Radiation Oncology and [2]Radiology, Dana-Farber Cancer Institute, Brigham and Women's Hospital, Harvard Medical School, Boston, MA, USA, [3]Radiation Oncology (MAASTRO), Research Institute GROW, Maastricht University, Maastricht, the Netherlands, [4]Machine Intelligence Unit, Indian Statistical Institute, Kolkata, India, [5]Department of Biostatistics & Computational Biology, Dana-Farber Cancer Institute, Boston, MA, USA, [6]Department of Radiation Oncology, Radboud University Medical Center, Nijmegen, the Netherlands

# Equal contribution

Subject areas:

Quantitative Imaging, Radiology, Radiomics, Cancer, Machine learning, Computational science

CORRESPONDING AUTHORS

Hugo Aerts, PhD
Dana-Farber Cancer Institute, Brigham and Women's Hospital, Harvard Medical School
450 Brookline Ave, JF518, Boston,
MA 02115-5450, P - 617.525.7156,
F - 617.582.6037
Email: Hugo_Aerts@dfci.harvard.edu

Chintan Parmar, MTech.
Dana-Farber Cancer Institute
450 Brookline Ave, JF518, Boston,
MA 02115-5450,
P - 617.525.7156,
F – 617.582.6037
Email:Chintan_Parmar@dfci.harvard.edu

Supplementary A: Datasets

This supplementary information contains the detailed description of the used datasets. It should be noted that the datasets has been previously used and described by Aerts et. al[1]. In order to enhance the redability of this manuscript, here we have reproduced the datasets description from the previous study of Aerts et. al[1]

Lung 1. MAASTRO NSCLC dataset

MAASTRO Clinic, (Maastricht, The Netherlands)

Patient population

Four hundred and twenty-two consecutive patients were included (132 women and 290 men), with inoperable, histologic or cytologic confirmed NSCLC, UICC stages I-IIIb, treated with radical radiotherapy alone (n = 196) or with chemo-radiation (n = 226). Mean age was 67,5 years (range: 33–91 years). The institutional review board approved the study. All research was carried out in accordance with Dutch law.

Treatment

During the study period, induction chemotherapy was standard of care for patients with N2/N3 and T4 tumors and consisted of three courses of gemcitabine (1,250 mg/m2 on days 1 and 8) in combination with cisplatin (75 mg/m2) or carboplatin (area under the concentration-time curve [AUC] 5) on day 1. Cycles were repeated every 21 days, and standard dose-reduction rules were applied. An interval between chemotherapy and start of radiotherapy of at minimum 14 days was mandatory.

All patients received an FDG PET-CT scan for radiotherapy treatment planning, in radiotherapy position on a dedicated PET-CT simulator with both arms above the head. For the FDG PET-CT scans a Siemens Biograph (SOMATOM Sensation-16 with an ECAT ACCEL PET scanner) was used. An intravenous injection of (weight * 4 + 20) MBq FDG (Tyco Health Care, Amsterdam, The Netherlands) was followed by 10 ml physiologic saline. After a 45-min uptake period, during which the patient was encouraged to rest, PET and CT images were acquired. A spiral CT (3 mm slice

thickness) with or without intravenous contrast was performed covering the complete thoracic region.

Radiotherapy planning was performed on a XiO (Computerized Medical Systems, St Louis, Missouri) treatment planning system, based on a convolution algorithm using inhomogeneity corrections.

Delineation based on fused PET-CT images was performed by the radiation oncologist by using a standard clinical delineation protocol. The protocol included fixed window level settings of both CT (lung W1700; L–300, mediastinum W600; L40) and PET scan (W30000; L15000) to be used for delineation. For all patients, a gross tumor volume (GTV) was defined based on FDG PET-CT data.

For patients treated with radical radiotherapy, the radiation dose was escalated to an individualized maximal total tumor dose, applying a mean lung dose of 19 Gy while respecting a maximum spinal cord dose of 54 Gy5. The maximal total tumor dose allowed was 79.2 Gy. There were no esophageal dose constraints. Radiotherapy was delivered twice a day in fractions of 1.8 Gy, 5 days per week, with a minimum of 8 h 27 between the two fractions. This protocol was applied as well in patients that received sequential chemo-radiation (n = 104).

Patients that received concurrent chemo-radiation (n = 100), were treated following 2 cycles of carboplatin-gemcitabine, a radiation dose of 45 Gy, in fractions of 1.5 Gy delivered twice a day for the first course, directly followed by an individualized dose ranging from 6 – 24 Gy and delivered in 2.0 Gy fractions once a day. In all patients, individualized patient dosimetry using electronic portal imaging devices was performed.

Lung 2. Radboud NSCLC Dataset

Radboud University Nijmegen Medical Center.

Patient population

This dataset included 225 consecutive patients with confirmed NSCLC (mean age, 65.5 years; range, 36–86 years), stages (I-IVa), treated at the Radboud University Nijmegen Medical Centre, The Netherlands, between February 2004 and October 2011.

Treatment

All primary tumors and the mediastinal N2 disease were cytologically or histologically proven. All patients underwent diagnostic work-up, including contrast enhanced CT of the thorax and upper abdomen, whole body 18F-FDG-PET/CT, MRI of the brain, bronchoscopy with transbronchial needle aspiration (TBNA), and/or oesophageal ultrasound fine needle aspiration (EUS-FNA) and/or endobronchial ultrasound with TBNA (EBUS-TBNA) and mediastinoscopy in case of PET-positive, cytologically negative mediastinal lymph nodes. After work up, all patients were discussed in a thoracic oncology multidisciplinary board. Prior to radiotherapy a CT of the thorax was performed in radiotherapy position for radiotherapy planning.

Patients in good general condition were treated with concurrent chemo radiotherapy, those with a contraindication for chemotherapy were treated by radiation alone, and all remaining patients were treated with a sequential chemotherapy and radiotherapy. The planned radiation dose to the primary tumor and metastatic mediastinal lymph nodes using CRT until March 2008 and IMRT afterwards, was 66Gy in 33 fractions delivered five times per week. Chemotherapeutic agents in the sequential regimen typically consisted of three courses of gemcitabine (1250mg/m2; on day 1 and 8) and cisplatinum (80mg/m2; on day 1). The concurrent schedules varied between referring hospitals; in Radboud University Nijmegen Medical Centre it consisted of two courses of etoposide (100mg/m2; on day 1–3) and cisplatinum (50mg/m2; on day 1 and 8), in Canisius-Wilhelmina Hospital one course of gemcitabine/cisplatinum was

administered prior to irradiation and two courses of etoposide/cisplatinum concurrently with radiation therapy. All research was carried out in compliance with the Helsinki Declaration and in accordance with Dutch law. The Institutional Review Board of the Radboud University Medical Center (RUMC) waved review due to the retrospective nature of this study. Follow-up was performed according to national guidelines.

Supplementary B: Feature Selection Methods

In literature, feature selection methods are mainly divided into three categories: filter methods, wrapper methods and embedded methods. Wrapper and embedded methods are classifier dependent approaches, whereas filter methods are classifier independent. Wrapper methods are basically the search methods, which search through the whole feature space and identify a relevant and non-redundant feature subset. Training/validation accuracy of a particular classifier (or model) is used as a measure of utility for the candidate feature subset. These computationally expensive methods may produce feature subsets that are overly specific to the classifiers and hence has low generalizability. Embedded methods incorporate feature selection as a part of training process and are computationally efficient as compared to the wrappers. However, they still use a quite strict model (classifier) structure assumption and hence lacks in the generalizability. On contrary, classifier-independent filter methods are the simple feature ranking methods based on some heuristic scoring criterion. Filters are computationally efficient and they have high generalizability and scalability. Therefore, here in this study we only used some popular filter based approaches for feature selection.

The defining component of filter based feature selection methods is the scoring/selection criterion, which is often known as 'relevance index'. All filter based feature selection methods can be divided into two categories: univariate methods and multivariate methods. In case of univariate methods, the scoring criterion only considers the relevancy of features ignoring the feature redundancy, whereas multivariate methods investigate the multivariate interaction within features and the scoring criterion is a weighted sum of feature relevancy and redundancy. We formulated the feature selection problem as defined by brown et al[2].

Let J be the scoring criterion (relevance index), Y be the class labels, X be the set of all features, X_k be the feature to be evaluated and S be the set of already selected features.

Univariate Feature Selection methods

Fisher score (FSCR)

Fisher score[3] based feature selection method selects features such that the between class distance is maximized and the within class distance is minimized. The scoring criterion is defined as

$$J_{Fisher}(X_k) = \frac{\sum_{m=1}^{2} n_m \left(\mu_{k,m} - \mu_k\right)^2}{\sum_{m=1}^{2} n_m \sigma_{k,m}^2}$$

Where μ_k is the overall mean of feature X_k, n_m is the number of samples in m'th class, and $\mu_{k,m}$ and $\sigma_{k,m}^2$ is the mean and variance of feature X_k on m'th class.

Relief (RELF)

Relief[4] assumes p randomly sampled data instances and defines the scoring criterion as

$$J_{Relief}(X_k) = \frac{1}{2}\sum_{t=1}^{p} d(X_{t,k} - X_{NM(x_t),k}) - d(X_{t,k} - X_{NH(x_t),k})$$

Where $X_{t,k}$ is the value of instance x_t on feature X_k, $X_{NH(x_t),k}$ and $X_{NM(x_t),k}$ are the values on the k'th feature of the nearest point to x_t with the same and different class label respectively, and d(.) denotes the distance. Here, we used p=50.

T-test (t-score) (TSCR)

T-test based feature selection evaluates a feature using a t-score, which is defined as

$$J_{ttest}(X_k) = \frac{\mu_1 - \mu_2}{\sqrt{\frac{{\sigma_1}^2}{n_1} + \frac{{\sigma_2}^2}{n_2}}}$$

where μ_1, μ_2 and ${\sigma_1}^2$, ${\sigma_2}^2$ are the means and variances of the two classes on feature X_k, whereas $n_1$and n_2 correspond to the cardinality of the two classes.

Chi-square (CHSQ)

Chi-square score for a feature with r different values is defined as

$$J_{Chi-square}(X_k) = \sum_{i=1}^{r} \sum_{m=1}^{2} \frac{(n_{im} - \mu_{im})^2}{\mu_{im}}$$

Where n_{im} is the number of samples with I'th feature value in m'th class and

$$\mu_{im} = \frac{n_{*m} n_{i*}}{N}$$

here, n_{i*} is the number of samples with i'th feature value, n_{*m} is the number of samples in class m and N is the number of samples.

Wilcoxon (WLCX)

Willcoxon is a non-parametric method based on ranks for the comparison of the population medians of the two classes. The scoring function is defined as

$$J_{Wilcoxon}(X_k) = (N-1) \frac{\sum_{m=1}^{2} n_m (\mu r_m - \mu r)^2}{\sum_{m=1}^{2} \sum_{i=1}^{n_m} (r_{mi} - \mu r)^2}$$

Where N is the total number of samples, n_m is the number of samples in class m, r_{mi} is the rank of sample i in class m, μr_m is the average rank of samples belonging to class m, and μr is the average rank of all samples.

Gini index (GINI)

For gini index, the scoring criterion is defined as

$$J_{gini}(X_k) = 1 - (\sum_{m=1}^{2} [p(m|X_k)]^2)$$

Where $p(m|X_k)$is the conditional probability of a class m given the feature X_k. Smaller the values of gini index correspond to higher feature relevance.

Mutual information maximization (MIM)

Mutual information maximization[5] uses an information theory to measure the relevance of a feature. The scoring criterion is defined as a mutual information between a feature and class labels. It is given as

$$J_{mim}(X_k) = I(X_k; Y)$$

Multivariate Feature Selection methods

Mutual information feature selection (MIFS)

Battiti et. al[6] proposed this multivariate feature selection method, which tries evaluate features based on their relevance with the class labels and penalizes the feature redundancy. The scoring criterion is a weighted sum of feature relevancy and redundancy and is given by

$$J_{mifs}(X_k) = I(X_k; Y) - \beta \sum_{X_j \in S} I(X_k; X_j)$$

Here the first term $I(X_k; Y)$ is the mutual information between the feature X_k and class labels, which indicates the feature relevancy.

Second term $\sum_{X_j \in S} I(X_k; X_j)$ corresponds to the feature redundancy. So a feature is only going to get the high score if it is highly relevant to the class labels and also non-redundant to the set of already selected features S. β is the configurable parameter, which must be set experimentally. Battiti et. al. [6] experimentally found that $\beta = 1$ is often optimal.

Minimum redundancy maximum relevance (MRMR)

As similar to MIFS, minimum redundancy maximum relevance (MRMR)[7] also tries to evaluate feature using relevancy-redundancy tradeoff. Here the configurable parameter β is set as the cardinality of the set of selected features. Hence, the scoring criterion is defined as

$$J_{mrmr}(X_k) = I(X_k;Y) - \frac{1}{|S|}\sum_{X_j \epsilon S} I(X_k;X_j)$$

Conditional infomax feature extraction (CIFE)

Conditional infomax feature extraction (CIFE)[8] also tries to optimize relevancy-redundancy trade off. In the case of cife, the penalty term is added by one more term that is called as conditional redundancy. This term has an opposite sing to the penalty (redundancy) term, which indicates that correlated features will be given high score if they have strong class conditional dependence in a combined manner.

$$J_{cife}(X_k) = I(X_k;Y) - \sum_{X_j \epsilon S} I\left(X_k;X_j\right) + \sum_{X_j \epsilon S} I\left(X_k;X_j|Y\right)$$

Joint mutual information (JMI)

In the case of joint mutual information (JMI)[9], the scoring criterion is the mutual information between the class labels and the joint random variable X_kX_j and it given by

$$J_{jmi}(X_k) = \sum_{X_j \epsilon S} I\left(X_kX_j;Y\right)$$

Conditional mutual information maximization (CMIM)

Fleuret[10] proposed the conditional mutual information maximization (CMIM) criterion. Here, basically the scoring criterion is the mutual information between the candidate feature X_k and class labels Y conditioned on the set of already selected features S.

$$J_{cmim}(X_k) = min_{\ X_j \epsilon S}\left[I(X_k;Y|X_j)\right]$$

Interaction capping (ICAP)

As similar to CMIM, interaction capping (ICAP)[11] also has a non-linear scoring criterion that is defined as

$$J_{icap}(X_k) = I(X_k;Y) - \sum_{X_j \in S} \max[0, \{I(X_k;X_j) - I(X_k;X_j|Y)\}]$$

It can be observed from the equation that the penalty will be lower if the candidate feature X_k has strong pairwise class conditional dependence with the set of already selected features.

Double input symmetric relevance (DISR)

Double input symmetric relevance (DISR)[12] is the modification of the joint mutual information criterion. Here the joint mutual information is normalized with a joint entropy term. The criterion is defined as

$$J_{jmi}(X_k) = \sum_{X_j \in S} \frac{I(X_k X_j;Y)}{H(X_k X_j Y)}$$

Publicly available Matlab implementations[2,13] were used for the implementations of feature selection methods. For further understanding of the theoretical assumptions and relations between these feature selection methods, we encourage reader to refer[2,13].

Supplementary C: Classification Methods

We used 12 classifiers belonging to different classifier families (Decision trees (DT), Boosting (BST), Discriminant analysis (DA), Bagging (BAG), Random forests (RF), Neural networks (Nnet), Generalized linear models (GLM), Nearest neighbors (NN), Partial least square and principle component regression (PLSR), Multiple adaptive regression splines (MARS), Bayesian (BY), Support vector machines(SVM)) in our analysis. In a recently published large comparative study, Fernandez-Delgado et. al[14] have evaluated 179 different classifiers arising from the 12 different families on 121 different data sets. This study has reported that most of the best performing classifiers of their study were implemented using R and tuned using the "caret" package[15]. We therefore chose R and caret as an implementation framework for our classifiers. A brief overview about implementation details of the classifiers and the corresponding parameters is given below. For the detailed theoretical description, we encourage reader to refer the individual method.

Decision tree (DT)

A C5.0 decision tree based classification method was used in the analysis. C5.0 function of the "C50" package was used for creating classification trees with default parameter tuning under caret interface.

Boosting (BST)

A Boosting ensemble of C5.0 decision tree was created using the R package "C50". Parameter tuning was carried out using caret interface. Number of boosting trials was varied in {1, 10, 20} with and without winnow.

Bayesian (BY)

R package "klaR" with default caret parameter tuning was used for the implementation of Naïve Bayes classifier.

Discriminant analysis (DA)

Flexible discriminant analysis is a non-linear extension of linear discriminant analysis. R package "mda" was used for the implementation. Parameter tuning was done using caret with the parameter nprune varing from 2:3:15 (2 to 15 with an increment of 3).

Bagging (BAG)

Bagging falls into the category of ensemble algorithms in machine learning. R package "ipred" was used for the implementation and default parameter tuning was done using caret interface.

Random forest (RF)

Random forest provides an improvement to bagging with a modification step of random sampling of predictors. R package "randomForest" with caret interface was used for the implementation. Parameter ntree was set to 500 and mtry was varied with values 2:3:29 (2 to 29 with an increment step of 3)

Neural network (Nnet)

Neural network, also known as multi layer perceptron is a non-linear classification model. It was implemented by a caret interface and R package "nnet" by tuning the size and weight decay parameter with values 1:2:9 and {0, 0.1, 0.01, 0.001, 0.0001} respectively.

Support vector machine (SVM)

SVM, with Gaussian kernel function was implemented using a caret interface and R package kernlab. Cost parameter C was varied with values $\{2^{-2}, 2^{-1}, 1, 2^{1}, 2^{2}\}$ and the parameter kernel spread was varied with values in $\{10^{-2}, 10^{-1}, 1, 10^{1}, 10^{2}\}$.

Nearest neighbor (NN)

K-nearest neighbor was implemented using the “knn” R package and caret interface. 10 different values of number of neighbors 5:2:23 (5 to 23 with an increment step of 2) were used.

Partial least squares and principal component regression (PLSR)

We used mvr function in “pls” package to fit PLSR model. Parameter tuning was done using caret. 10 different values of the number of components 1:1:10 were used.

Generalized linear models (GLM)

A generalized linear model via penalized maximum likelihood was fitted using the glmnet function of the R package “glment” and the default parameter tuning with caret interface.

Multivariate adaptive regression splines (MARS)

An additive MARS model was built using the gcvEarth function of the R package “earth” with default parameter tuning using caret interface.

Supplementary Figures

Figure S1 | Predictive performance (AUC) of feature selection (in rows) and classification methods (in columns) with top 10 selected features.

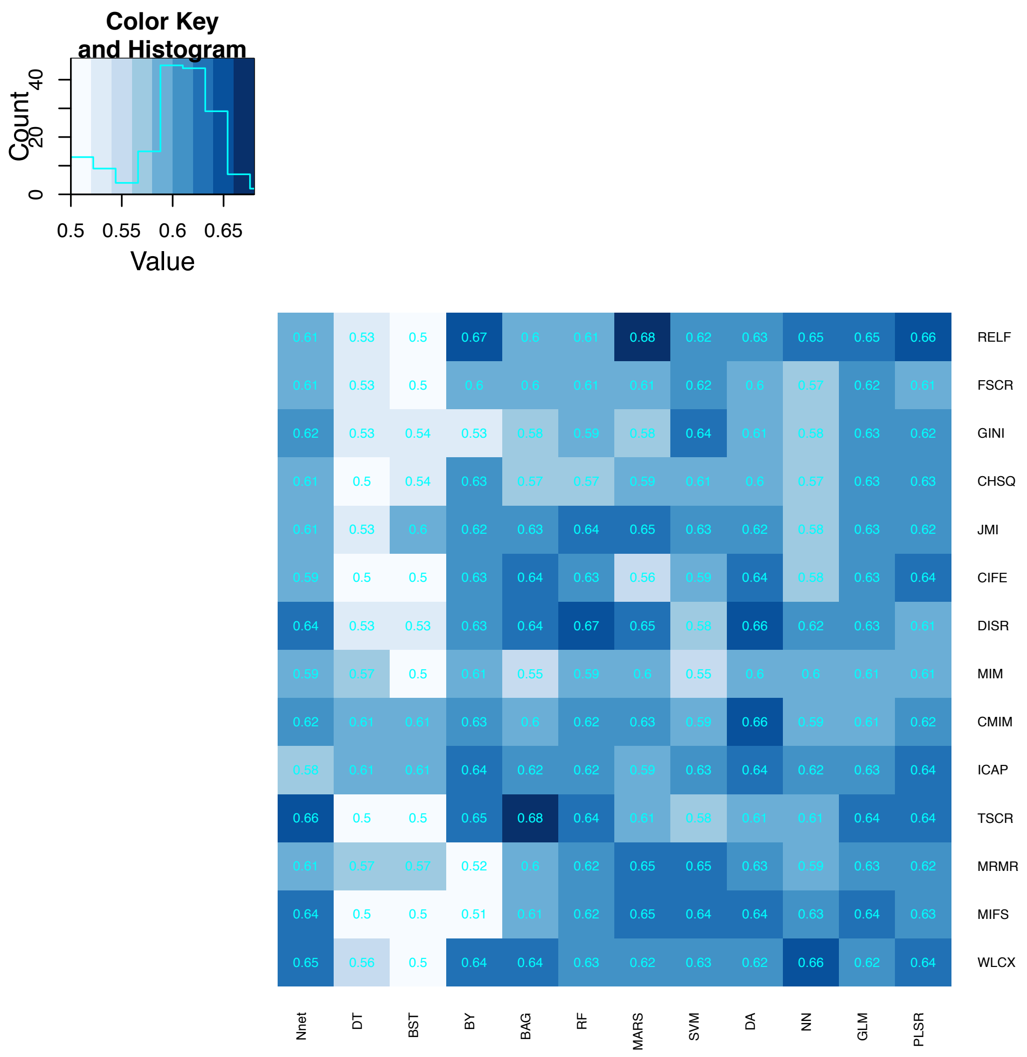

Figure S2 | Predictive performance (AUC) of feature selection and classification methods with top 20 selected features.

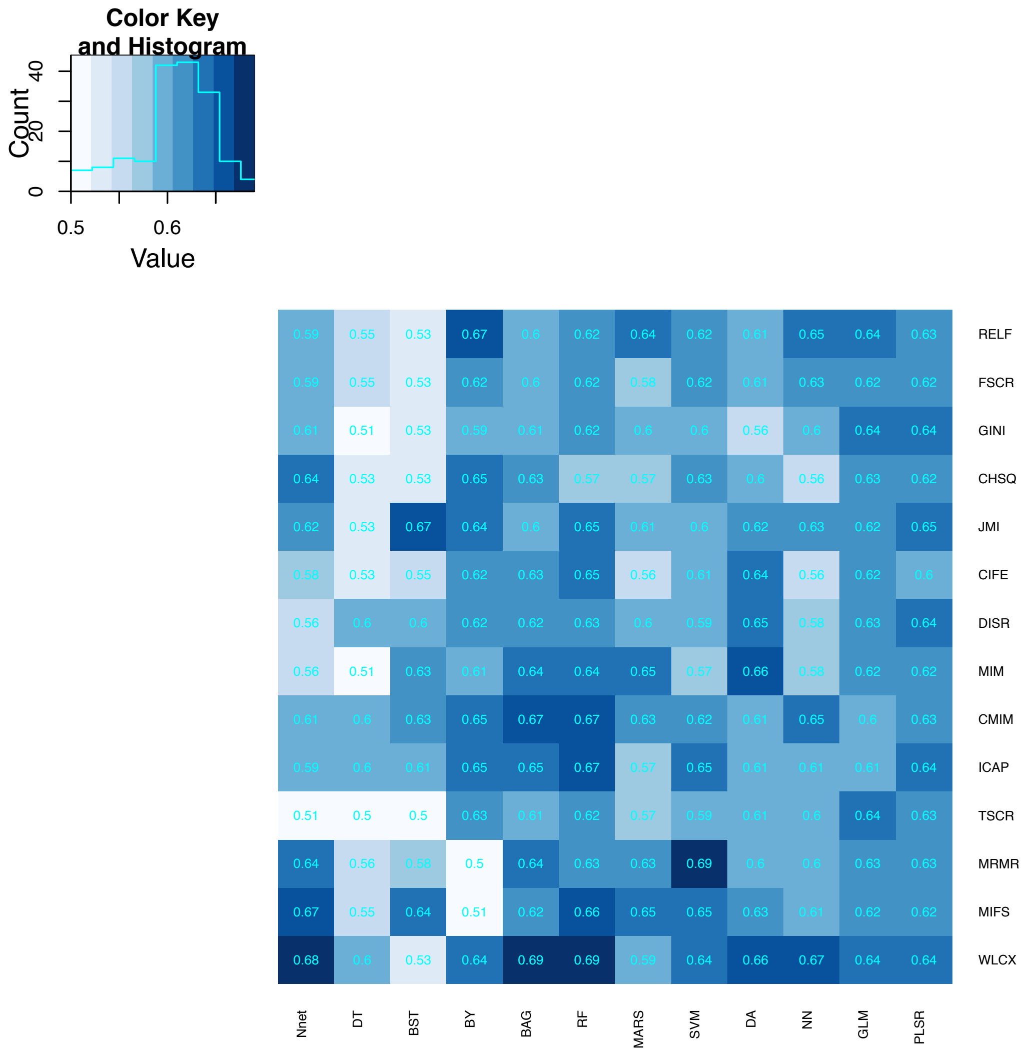

Figure S3 | Predictive performance (AUC) of feature selection and classification methods with top 40 selected features.

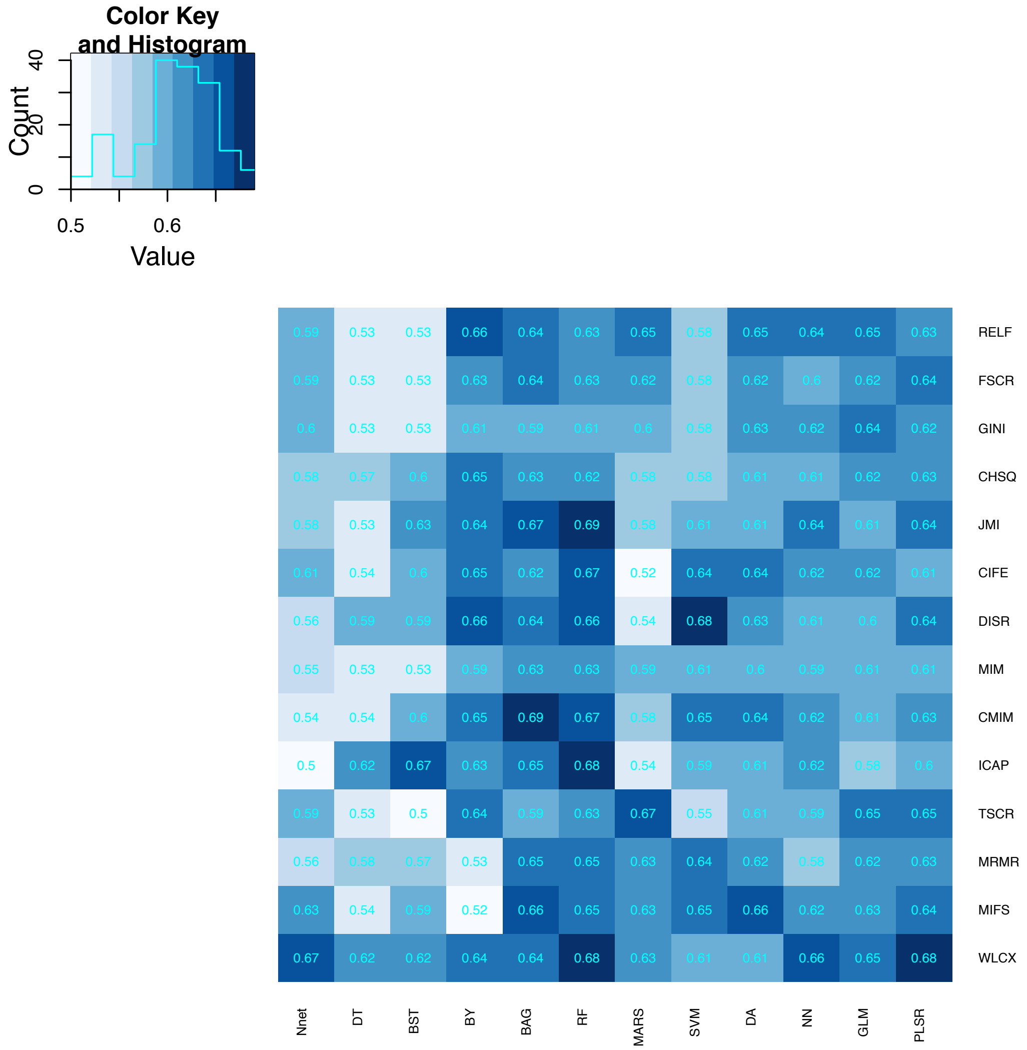

Figure S4 | Predictive performance (AUC) of feature selection and classification methods with top 50 selected features.

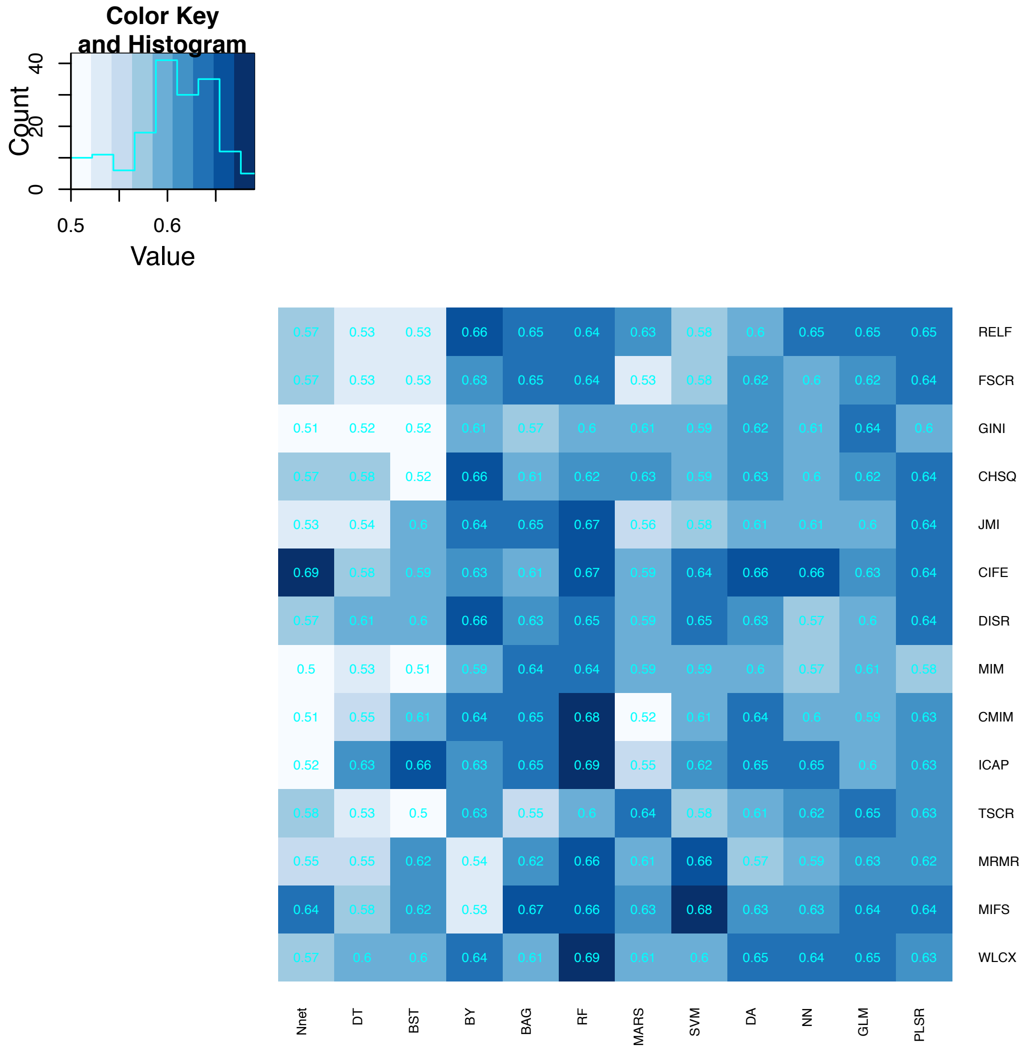

Figure S5 | Predictive performance (median over all feature selection methods) corresponding to classification methods (in columns) and the number of selected features (in rows).

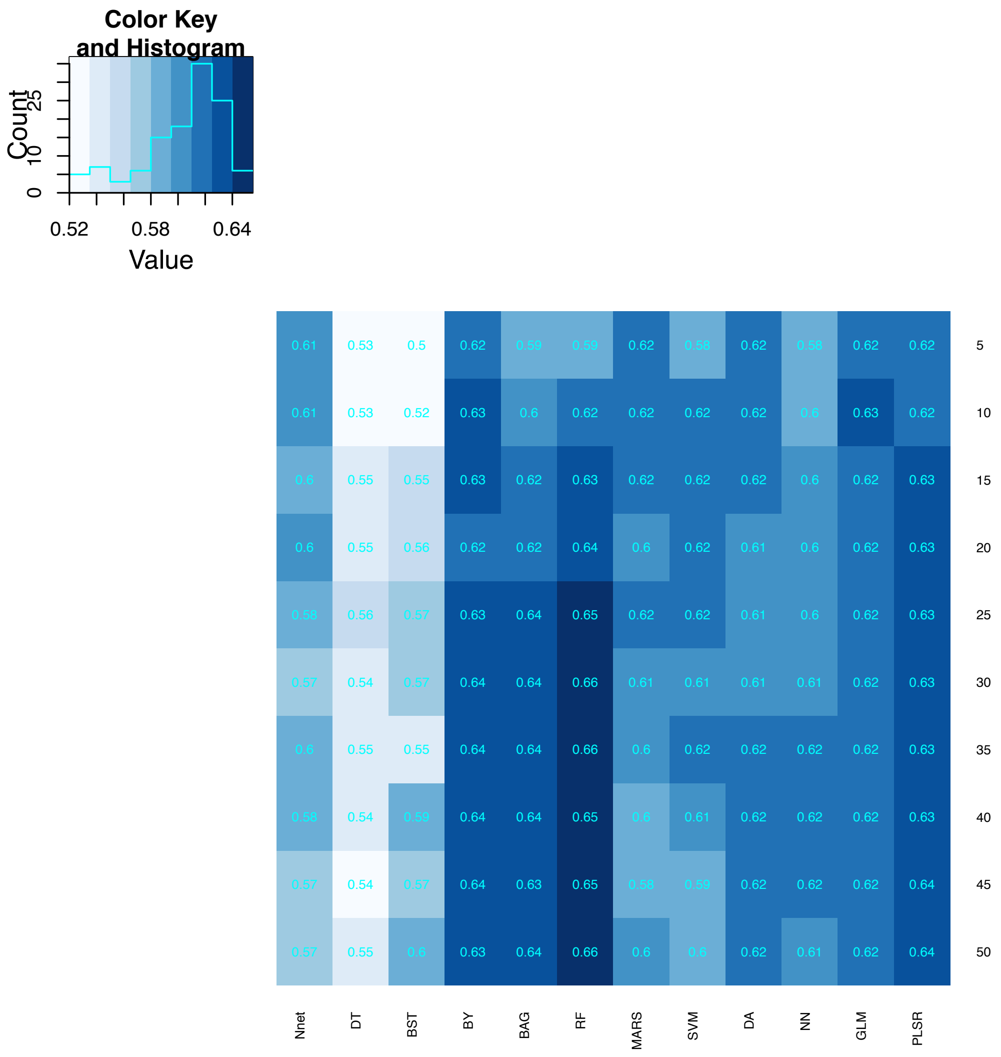

Figure S6 | Predictive performance (median over all classification methods) corresponding to feature selection methods (in rows) and the number of selected features (in columns).

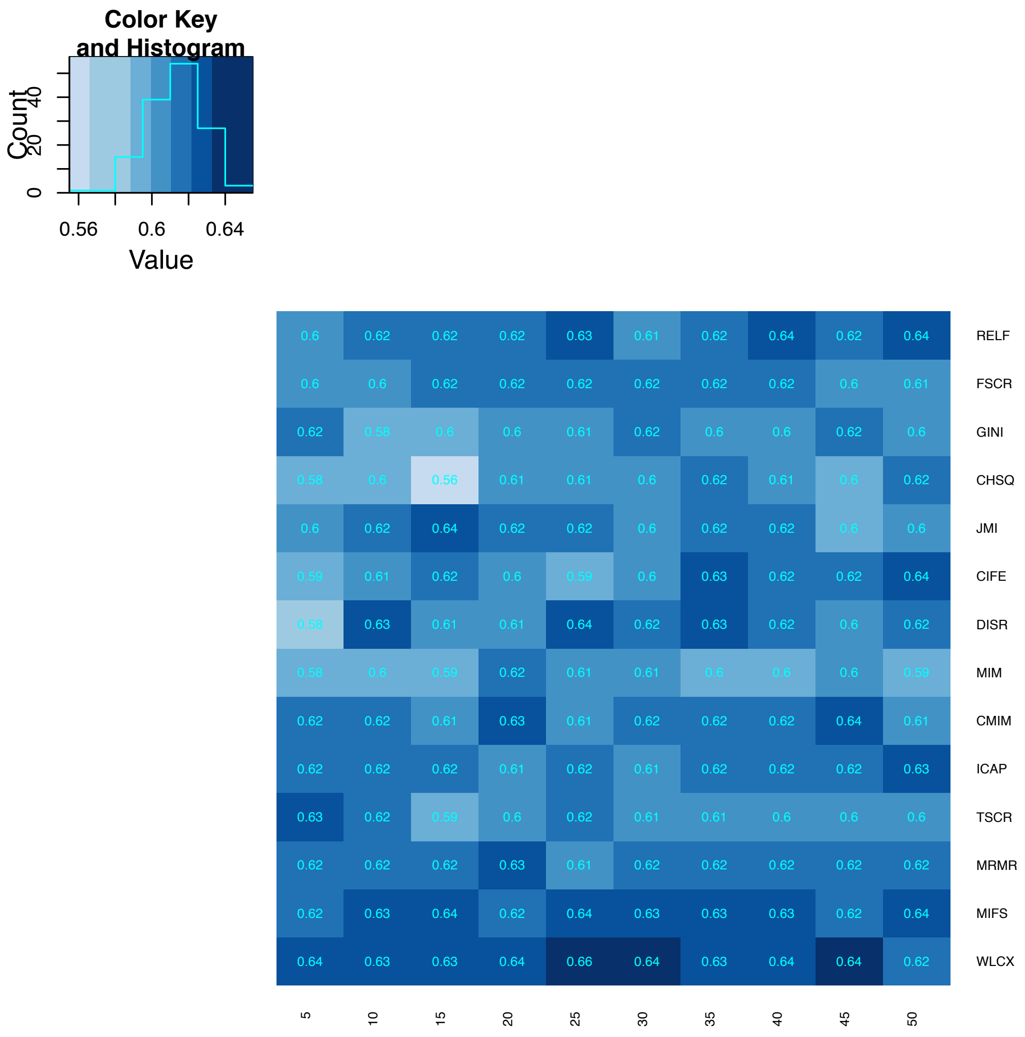

Figure S7 | Predictive performance (median over the number of selected features) corresponding to classification methods (in columns) and feature selection methods (in rows).

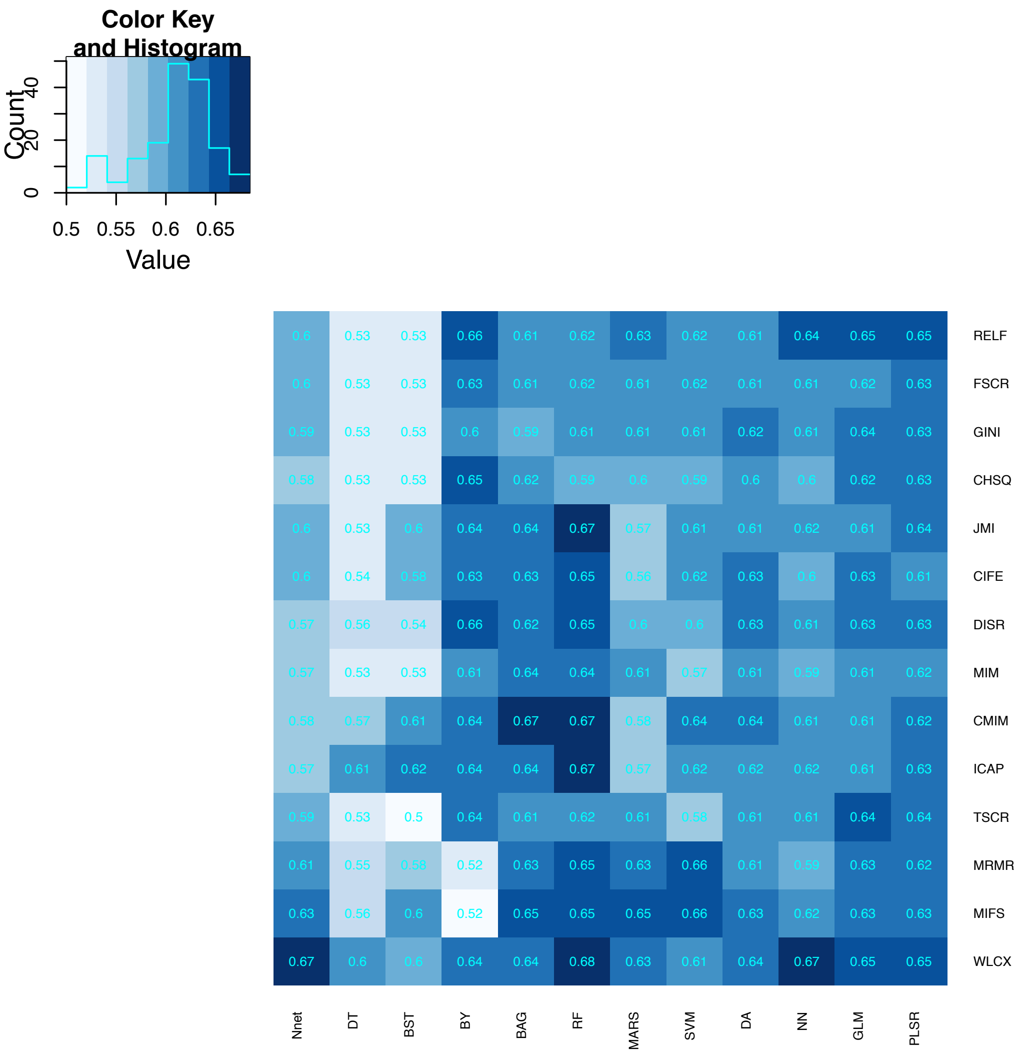

REFERENCES

1 Aerts, H. J. *et al.* Decoding tumour phenotype by noninvasive imaging using a quantitative radiomics approach. *Nature communications* **5** (2014).

2 Brown, G., Pocock, A., Zhao, M.-J. & Luján, M. Conditional likelihood maximisation: a unifying framework for information theoretic feature selection. *The Journal of Machine Learning Research* **13**, 27-66 (2012).

3 Duda, R. O., Hart, P. E. & Stork, D. G. *Pattern classification*. (John Wiley & Sons, 2012).

4 Kira, K. & Rendell, L. A. in *Proceedings of the ninth international workshop on Machine learning.* 249-256.

5 Lewis, D. D. in *Proceedings of the workshop on Speech and Natural Language.* 212-217 (Association for Computational Linguistics).

6 Battiti, R. Using mutual information for selecting features in supervised neural net learning. *Neural Networks, IEEE Transactions on* **5**, 537-550 (1994).

7 Peng, H., Long, F. & Ding, C. Feature selection based on mutual information criteria of max-dependency, max-relevance, and min-redundancy. *Pattern Analysis and Machine Intelligence, IEEE Transactions on* **27**, 1226-1238 (2005).

8 Lin, D. & Tang, X. in *Computer Vision–ECCV 2006* 68-82 (Springer, 2006).

9 Yang, H. H. & Moody, J. E. in *NIPS.* 687-702 (Citeseer).

10 Fleuret, F. Fast binary feature selection with conditional mutual information. *The Journal of Machine Learning Research* **5**, 1531-1555 (2004).

11 Jakulin, A. *Machine learning based on attribute interactions*, Univerza v Ljubljani, (2005).

12 Meyer, P. E. & Bontempi, G. in *Applications of Evolutionary Computing* 91-102 (Springer, 2006).

13 Zhao, Z. *et al.* Advancing feature selection research. *ASU feature selection repository* (2010).

14 Fernández-Delgado, M., Cernadas, E., Barro, S. & Amorim, D. Do we need hundreds of classifiers to solve real world classification problems? *The Journal of Machine Learning Research* **15**, 3133-3181 (2014).

15 Kuhn, M. Building predictive models in R using the caret package. *Journal of Statistical Software* **28**, 1-26 (2008).
